# Phenomics-derived temporal maize health and environmental index enhance physiology-informed genomic prediction of yield across environments

**DOI:** 10.1093/plphys/kiag344

**Published:** 2026-07-30

**Authors:** Alper Adak, Aaron J DeSalvio, Seth C Murray, James B Holland, Natalia De Leon, Martin O Bohn, Jode Edwards, David Ertl, Candice Hirsch, Shawn Kaeppler, Joseph E Knoll, Dayane C Lima, Richard Minyo, James C Schnable, Rajandeep S Sekhon, Maninder P Singh, Erin E Sparks, Addie Thompson, Mitchell R Tuinstra, Jacob D Washburn, Teclemariam Weldekidan

**Affiliations:** Department of Soil and Crop Sciences, Texas A&M University, College Station, TX 77843-2474, United States; Interdisciplinary Graduate Program in Genetics and Genomics (Department of Biochemistry and Biophysics), Texas A&M University, College Station, TX 77843-2128, United States; Department of Soil and Crop Sciences, Texas A&M University, College Station, TX 77843-2474, United States; United States Department of Agriculture, Agricultural Research Service Plant Science Research Unit, Raleigh, NC 27695, United States; Department of Crop and Soil Sciences, North Carolina State University, Raleigh, NC 27695, USA; Department of Plant and Agroecosystem Sciences, University of Wisconsin–Madison, 1575 Linden Drive, Madison, WI 53706, United States; Department of Plant and Agroecosystem Sciences, University of Wisconsin–Madison, 1575 Linden Drive, Madison, WI 53706, United States; USDA-ARS Corn Insect and Crop Genetics Research Unit, Ames, IA 50011-1051, United States; Iowa Corn Promotion Board, Johnston, IA 50131, United States; Department of Agronomy and Plant Genetics, University of Minnesota, St. Paul, MN 55108, United States; Department of Crop and Soil Sciences, North Carolina State University, Raleigh, NC 27695, USA; USDA-ARS Crop Genetics and Breeding Research Unit, Tifton, GA 31793, United States; Department of Crop and Soil Sciences, North Carolina State University, Raleigh, NC 27695, USA; Department of Horticulture and Crop Science, College of Food, Agricultural, and Environmental Sciences, The Ohio State University, Wooster, OH 44691, United States; Department of Agronomy and Horticulture, University of Nebraska–Lincoln, Lincoln, NE 68588, United States; Department of Genetics and Biochemistry, Clemson University, Clemson, SC 29634, United States; Department of Plant, Soil and Microbial Sciences, Michigan State University, East Lansing, MI 48824, United States; Department of Plant and Soil Sciences, University of Delaware, Newark, DE 19716, United States; Department of Plant, Soil and Microbial Sciences, Michigan State University, East Lansing, MI 48824, United States; Department of Agronomy, Purdue University, West Lafayette, IN 47907, United States; USDA-ARS, Plant Genetics Research Unit, Columbia, MO, United States; Department of Plant and Soil Sciences, University of Delaware, Newark, DE 19716, United States

## Abstract

Integrating multi-omics data, including phenomic, genomic, and environmental inputs, offers a powerful approach for enhancing maize performance and predicting grain yield. In this study, crop health was quantified using temporal NGRDI (Normalized Green Red Difference Index) trajectories collected from 16 unoccupied (unmanned) aerial vehicle or system (UAV or UAS, drones and sensors) flights (from 19 to 117 d after planting) in maize trials conducted in Texas. Crop health indices (CHIs) were calculated through area under the curve (CHI_AUC_) and functional principal component analysis (CHI_FPCA_), capturing dynamic plant health responses throughout the growing season. Heritability estimates of NGRDI fluctuated between 0.3 and 0.7, averaging 0.51 ± 0.02, reflecting consistent genetic contributions to growth dynamics. CHIs derived from a favorable (irrigated) trial in Texas effectively separated high- and low-yielding hybrids across 41 environment-tester combinations, achieving significant differentiation in 27 (CHI_AUC_﻿) and 28 (CHI_FPCA1_) environments. In comparison, only 21 environments were differentiated when using grain yield alone. Genomic mapping of temporal NGRDI revealed key quantitative trait loci (QTLs) linked to maize growth, containing candidate genes including *br2*, *phyC1*, *wus1*, *mads69*, *cct1*, *rap2*, *miR172*, and *gl15*, associated with canopy development, flowering regulation, and drought adaptation. Integrating multi-omics data into phenomic- and environment-informed genomic prediction models improved yield prediction accuracy by approximately 18.5%, particularly for untested genotypes in both tested and untested environments. These findings demonstrate that multi-omics integration provides a scalable framework for enhancing maize performance and advancing grain yield prediction across diverse agricultural systems.

## Teaser

Integrating temporal phenomics, genomics, and environmental data improved maize yield prediction by ∼18.5% and identified key loci underlying growth, development, and stress response.

## Introduction

Understanding the resilience of maize hybrids, the maintenance of plant health during development and despite stress, across multiple environments is critical for breeding programs aimed at enhancing crop grain yield stability. High-throughput phenotyping technologies have revolutionized the way we quantify complex traits, such as plant growth and health dynamics and the responsiveness of genotypes to diverse environments over time and growth (Cobb et al. 2013; Chowhan and Chakraborty 2022). These technologies, combined with appropriate statistical methods, allow researchers to extract valuable metrics like the area under the curve (AUC) of growth trajectories and functional principal component analysis (FPCA) scores, which are becoming increasingly descriptive and predictive in modern crop science (Araus et al. 2018; Miao et al. 2020; Adak et al. 2024).

Assessing crop health through high-throughput field phenotyping (HTFP) marks an important frontier in plant biology, plant breeding, and quantitative genetics (Singh et al. 2016, 2021; Tardieu et al. 2017; Ghosal et al. 2018). HTFP combines modern technologies with an understanding of plant genetics and environmental interactions, enabling researchers to study crop health and growth in changing conditions. By employing state-of-the-art sensors, imaging systems, and data analytics, researchers can capture a vast array of phenotypic traits in near real-time, shedding light on how different crop varieties respond to diverse stressors (Naik et al. 2017; Herr et al. 2023). This comprehensive assessment helps identify key genetic factors influencing growth, which can guide the breeding of crops with better adaptability and fitness. The integration of HTFP with quantitative genetics holds great potential for advancing our understanding of genetic pathways, improving breeding strategies, and promoting the development of resilient cultivars for sustainable and secure global food production.

Previous studies have highlighted the application of unoccupied aerial vehicles (UAVs, also called UAS or drones) in quantifying crop responsiveness to biological and environmental stresses during growth, providing a foundation for the diverse UAV-based applications described below. RGB-derived traits have been successfully used to track drought resistance in rice, and UAV time-series imagery has revealed relationships between heat stress and specific maize growth phases, including the identification of candidate genes controlling heat shock proteins (Jiang et al. 2021; Adak et al. 2023). Similarly, UAV-based thermal imaging has been effective in identifying Fusarium head blight in wheat and has shown potential for detecting plant stress before visible symptoms emerge (Francesconi et al. 2021; Stutsel et al. 2021). UAV imagery has also been employed to monitor drought stress in wheat and dry beans, helping to differentiate between environmental stressors and identify high-yielding varieties (Sankaran et al. 2018; Bhandari et al. 2021). Moreover, machine learning models using UAV data have demonstrated high accuracy in classifying stress responses, such as slow- and fast-wilting soybean varieties under drought conditions (Zhou et al. 2020). However, these approaches have often been constrained by limited temporal data and sparsity in flight dates, restricting their ability to provide a comprehensive understanding of temporal plant responses across different trials. Moreover, critical gaps remain regarding the relationship between UAV-derived crop Health Indices (CHI) and actual yield performance, particularly in multi-environment trials.

In this study, we utilized 250 maize hybrid genotypes from the Genomes to Fields (G2F; https://www.genomes2fields.org/) multi-environment project, evaluated under irrigated and nonirrigated conditions in Texas in 2020, to develop a quantitative framework for measuring crop health trajectories as indicators of plant growth using AUC and FPCA. Using a UAV equipped with an RGB camera, we conducted repeated aerial surveys throughout plant development. Temporal trajectories of the vegetation index NGRDI (Normalized Green–Red Difference Index) derived from these surveys exhibited substantial variation across trial conditions and were used to calculate CHIs (integrative, nonspecific indicators of overall plant health and physiological fitness across growth) based on AUC and FPCA scores. CHIs derived from 2 trials conducted in a single training environment were then associated with yield performance of these genotypes across diverse environments. The primary objective of this research was to develop and test new analytical approaches for quantifying heritable crop health using HTFP technology at 1 location and to assess their effectiveness in distinguishing high- and low-yielding hybrids across multiple environments. Additionally, quantitative trait loci (QTLs) linked to temporal variation of NGRDI were identified to suggest candidate genes which hint at underlying biological causes.

Through these methodologies and their application in maize, we aimed to enhance understanding and assessment of growth adaptability and performance stability, thereby contributing to more effective quantitative genetic and phenomic research and improved crop breeding strategies.

## Results

### Temporal analysis of NGRDI trajectories

**Figure 1:**
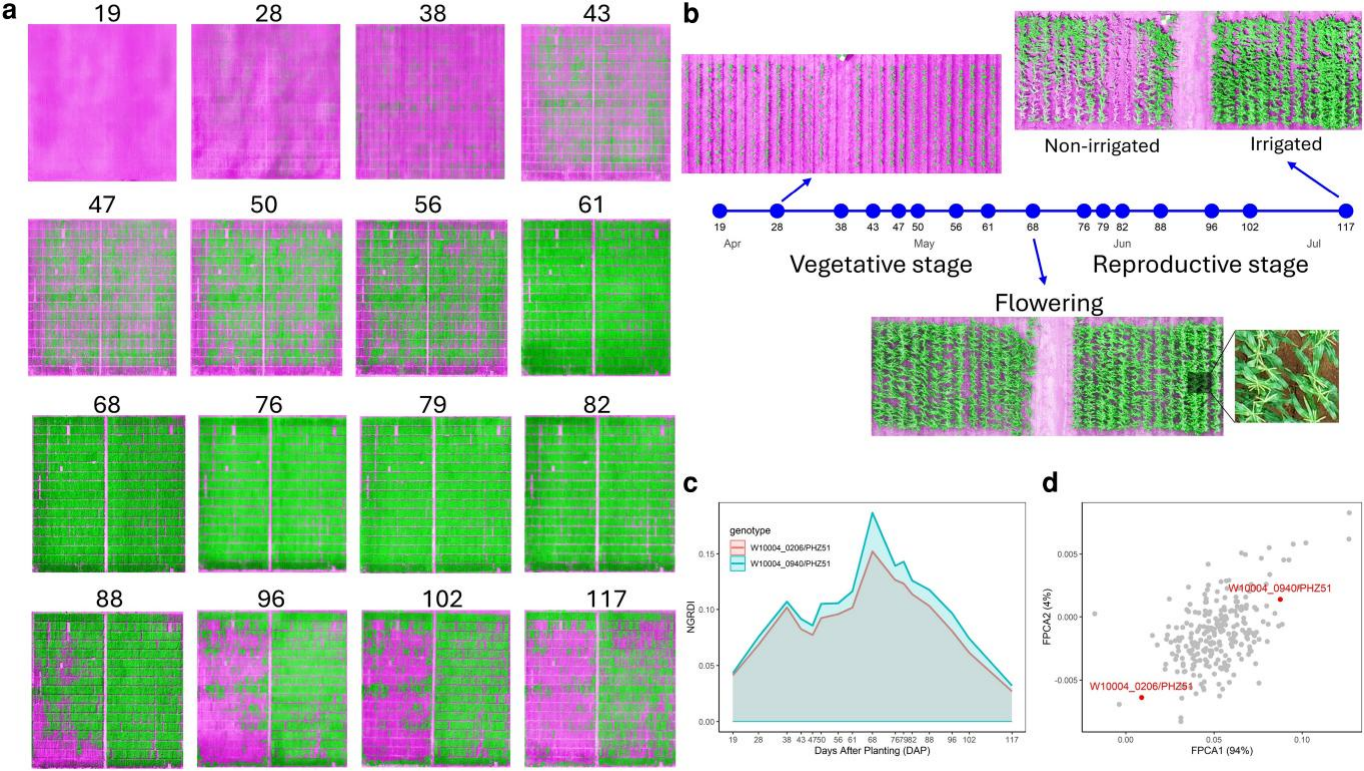
Visualization of growth of maize hybrids and phenotypic analysis of maize hybrids under irrigated and nonirrigated conditions across multiple time points. a) False-color images of maize plots captured by UAV at 16 different days after planting (DAP) representing each flight date. Each image represents the crop canopy condition at specific time points (19 to 117 DAP), illustrating canopy development and senescence progression under irrigated (right) and nonirrigated (left) trials. b) Maize growth stages are shown along the *x*-axis (days after planting), with blue dots connected by a line and separated into vegetative, flowering, and reproductive stages. Images on the right show a close-up comparison of nonirrigated and irrigated plots during the reproductive stage, highlighting differences in canopy cover due to water stress. An additional close-up image shows the flowering stage, with tassels in the enlargement shown in the inset. c) Area under the curve (AUC) of NGRDI values across 16 time points for 2 distinct hybrids, representing Crop Health Index (CHI_AUC_). d) FPCA1 and FPCA2 values, highlighting the same 2 hybrids (highlighted points and labels), also representing Crop Health Index CHI_FPCA_.

**Figure 2:**
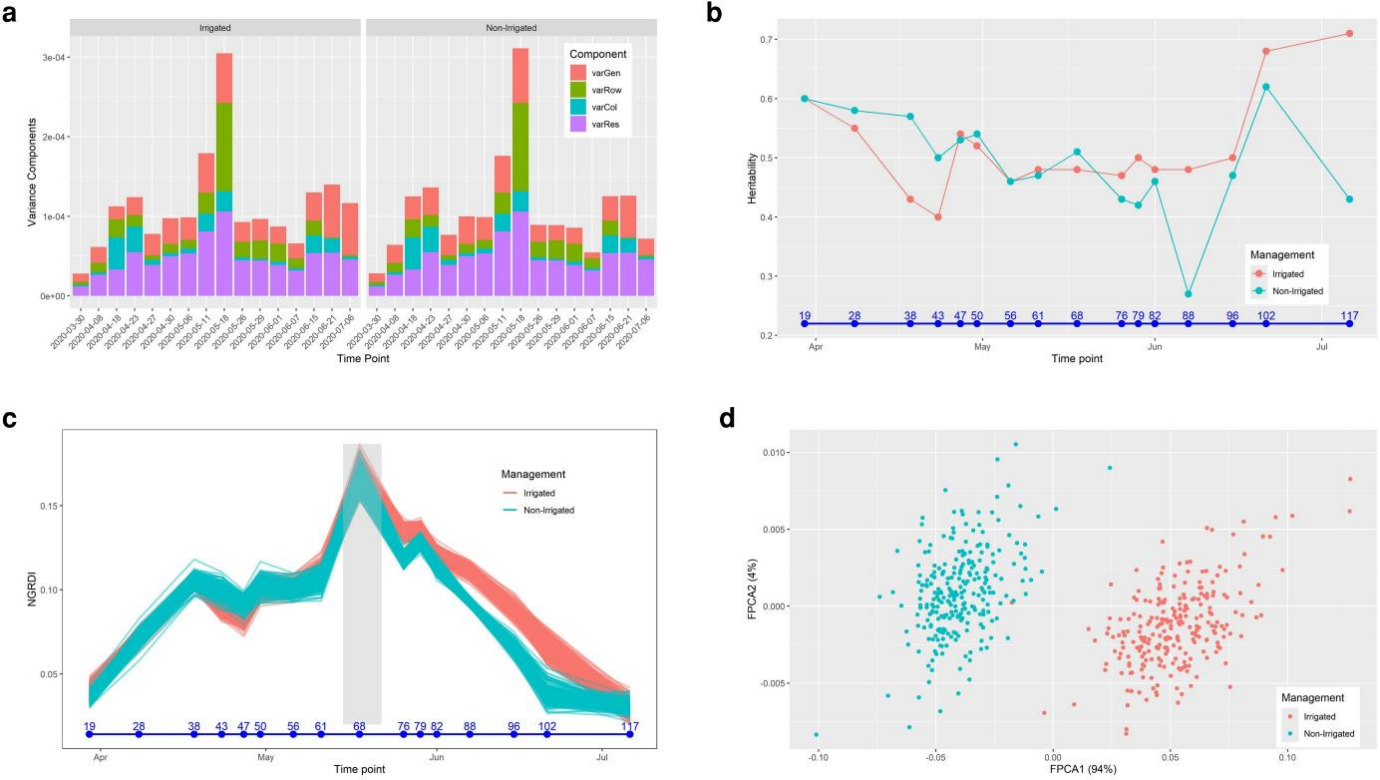
Variance decomposition, heritability, normalized green-red difference index (NGRDI) trajectory, and functional principal component analysis (FPCA) results for maize hybrids grown under Texas irrigated and nonirrigated conditions across multiple time points. a) Variance decomposition of NGRDI for each time point, partitioned by genetic (varGen), row and column components (varRow and varCol) constructing the RCDB design, and residual (varRes) components under irrigated and nonirrigated conditions. Flowering occurred around 2020-05-18 (yyyy-mm-dd) corresponding to 68 d after planting (DAP). b) Heritability estimates of NGRDI across UAV flight time points (days after planting, DAP), with dots representing individual time points along the *x*-axis (in days). c) Temporal trajectory of NGRDI under irrigated and nonirrigated conditions, with shaded areas representing flowering times. d) Scatterplot of the first 2 functional principal components (FPCA1 and FPCA2) explaining variance in NGRDI trajectories, differentiating irrigated and nonirrigated conditions.

Temporal NGRDI trajectories derived from repeated UAV flights (Fig. 1, a and b) were used for subsequent analyses. The variance decomposition of NGRDI across time points revealed significant differences in the contribution of genetic and spatial variation (row and column effects) under irrigated and nonirrigated conditions. The variation shows changes across the growth stages, with pronounced peaks around the mid-growing season. This trend was consistent across both management conditions, although the magnitude varied (Fig. 2a). The heritability estimates of NGRDI across time points were depicted, indicating fluctuation throughout the growth period to some extent (Fig. 2b). Mean heritability was slightly higher in the irrigated trial with 0.52 ± 0.08 than in the nonirrigated trial with 0.49 ± 0.09.

Notably, differences in the temporal trajectories of NGRDI between irrigated and nonirrigated trials became apparent after flowering and continued throughout the reproductive stages, when water stress was most severe (Fig. 2c). The FPCA analysis differentiated NGRDI trajectories between the 2 management conditions. FPCA1 captured the majority of the variation (94%), with a clear separation between irrigated and nonirrigated trials (Fig. 2d).

### Connecting CHIs to yield performance

In the current study, 3 hybrid populations were developed by crossing a common set of 220 inbred lines and subsequently top-crossing them with 3 different testers (PHZ51, PHK76, and PHP02). Phenotypic and phenomic data used for downstream analyses were obtained from multi-environment field trials conducted across 25 unique US environments during the 2020 and 2021 growing seasons as part of the Genomes to Fields (G2F) initiative (Lima et al. 2023).

The 25 environments (defined as location × year combinations) spanned a wide range of United States growing conditions. FPCA was used to summarize and visualize the distribution of environments based on the environmental index (THI/DL) evaluated from planting to 100 days after planting (DAP), while *k*-means clustering was applied to group the 25 environments into 2 main clusters with similar temporal environmental profiles (Figure S1).

**Figure 3:**
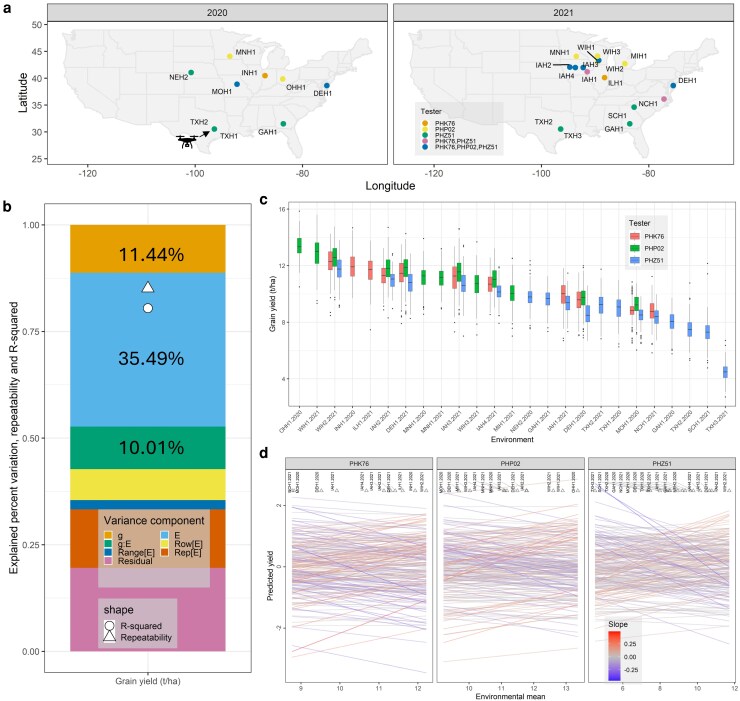
Geographic and statistical analysis of maize grain yield across different environments and testers. a) Maps of the United States showing the locations of trial environments across the 2020 and 2021 growing seasons. Testers used in the trials are color-coded, with each point representing a specific environment. The UAV icon in TXH1 and TXH2 indicates the environment of UAV phenotyping in 2020. b) Variance component analysis of grain yield using a Genotype-by-Environment (G × E) model (Eq. 2). The explained variation in grain yield (t/ha) is decomposed into genetic, environmental, and interaction components, with repeatability and R-squared values for each component indicated by different shapes. c) Box plots showing grain yield distribution for each environment (*x*-axis) across testers (PHK76, PHP02, PHZ51). Environments are organized by year and location, and testers are color-coded as shown in the legend. In each boxplot, the center line represents the median; the box limits indicate the interquartile range (25th and 75th percentiles); whiskers extend to 1.5× the interquartile range; and points beyond the whiskers represent outliers. d) Reaction norms by Finlay and Wilkinson regression for predicted yield across environments (ordered from left to right by increasing mean grain yield; environment names shown along the top, rotated by 90°), categorized by tester (PHK76, PHP02, PHZ51). The slope of each line indicates the genotype's response across the environmental gradient, with color indicating slope magnitude (steeper slopes in red, flatter slopes in blue). Grain yield (t ha^−1^) was centered and scaled on the *y*-axis.

The PHZ51 hybrid population was evaluated in 17 environments, while the PHK76 and PHP02 populations were evaluated in 11 and 13 environments, respectively (Fig. 3a). All integrative analyses, modeling, and interpretations presented here represent original results of the current study. G × E interaction accounted for 10.0% of the total variation in grain yield, while genotype and environment individually explained 11.4% and 35.5% of the variation, respectively (Fig. 3b). Hybrid performance exhibited both positive and negative deviations across environments, indicating bidirectional G × E responses along an increasing environmental yield gradient toward more favorable growing conditions. Accordingly, genotypic effects of hybrids were ordered based on the environmental mean grain yield (Fig. 3c). Finlay and Wilkinson regression further characterized hybrid–environment interactions by estimating the response (slope) of each hybrid across environments within each of the 3 hybrid populations (Fig. 3d).

**Figure 4:**
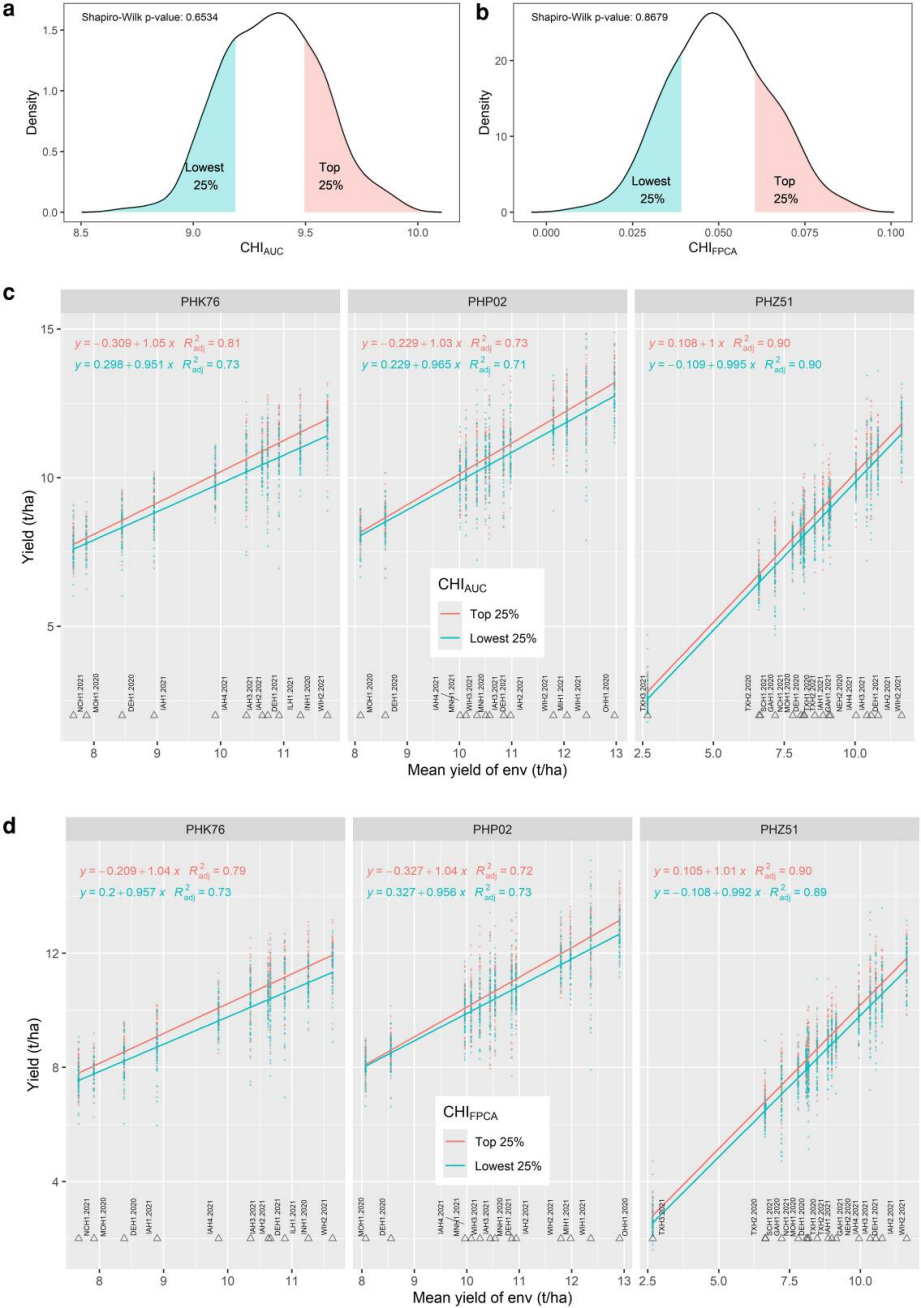
Application of 2 different Crop Health Indices (CHI) to grain yield across 3 hybrid populations and multi-environments. a) The distribution of CHI based on the area under the curve (CHI_AUC_), while b) presents CHI based on functional principal component analysis (CHI_FPCA_). Shapiro-Wilk *P*-values indicate the normality of the distributions. Lowest and highest 25th percentiles are shaded, representing hybrids in the lower and higher 25th quantiles of CHIs. Both CHI_AUC_ and CHI_FPCA_ were calculated only from the favorable environment,—the Texas irrigated trial in 2020 (TXH1.2020). c) Finlay-Wilkinson regression plots showing the relationship between mean environment yield and hybrids in the lower and higher CHI_AUC_ percentiles, while d) shows similar regression plots for hybrids in the lower and higher CHI_FPCA_ percentiles. Each plot is divided by tester type: PHK76, PHP02, and PHZ51, representing 3 different hybrid populations. Regression lines for lower and higher percentiles are shown with corresponding equations and R^2^ values indicating the fit for each percentile. The *x*-axis represents the mean yield of each environment (t/ha), and the *y*-axis represents each hybrid's yield (t/ha).

The 2 CHIs, CHI_AUC_, and CHI_FPCA_, captured systematic differences in grain yield performance across the 3 hybrid populations. Both indices exhibited approximately normal distributions (Fig. 4, a and b), supporting their use for percentile-based classification of hybrids.

Across all tester-based populations, hybrids in the upper CHI percentiles consistently achieved higher grain yield than those in the lower percentiles across environments (Fig. 4, c and d). This demonstrates that NGRDI CHIs calculated from a single environment retained strong discriminative power when applied to hybrids evaluated under diverse environmental conditions that lacked UAV data.

Finlay–Wilkinson regression analyses proved that the 2 CHI-defined groups exhibited highly similar slopes (≈ 0.95 to 1.05 across all panels), indicating comparable responsiveness to changes in environmental mean yield. Importantly, the regression lines for upper-percentile hybrids remained consistently above those of the lower-percentile group and did not cross across environments, confirming stable yield separation between groups along the environmental yield gradient. As a result, yield differences between groups became increasingly pronounced toward favorable, high-yielding environments. Together, these results show that CHI-based classification derived from temporal NGRDI data distinguishes high- and low-yielding hybrids primarily through differences in yield level rather than differential environmental sensitivity (Fig. 4, c and d).

**Figure 5:**
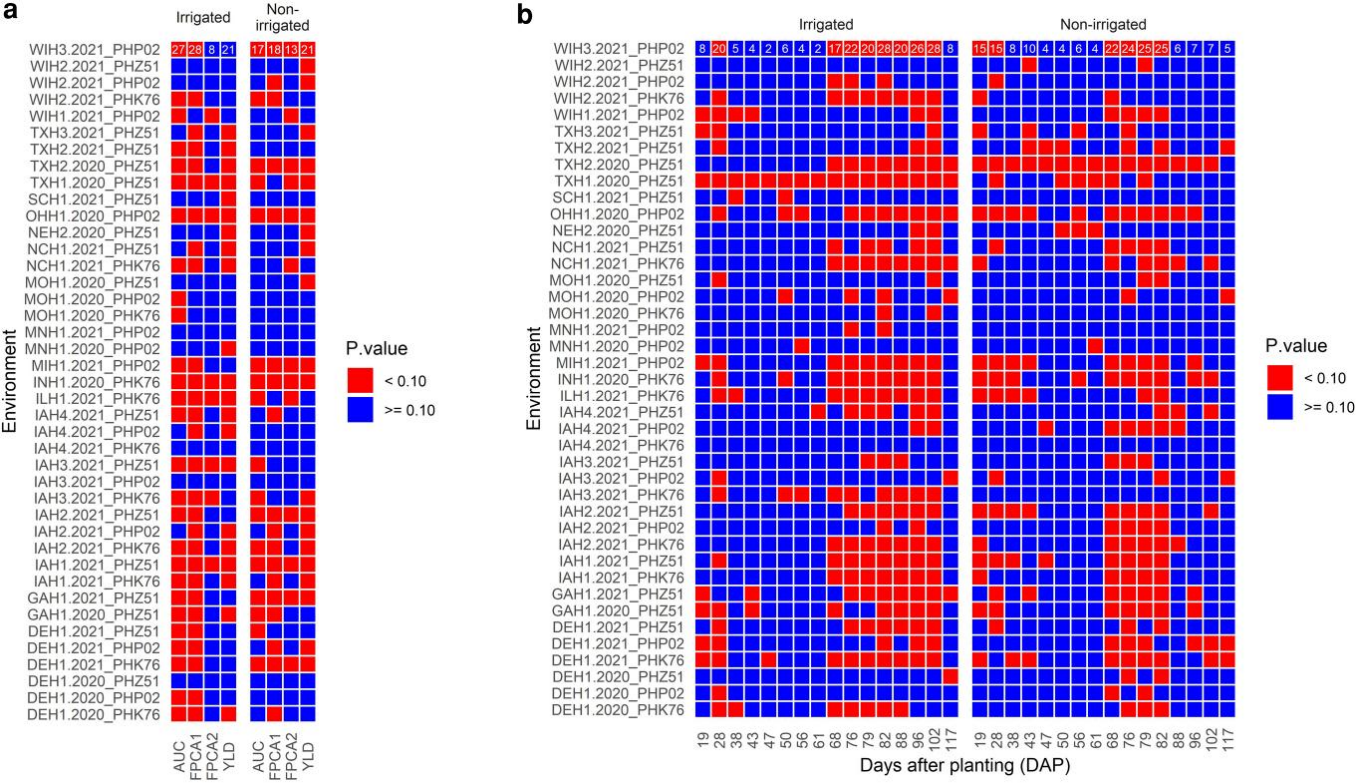
Significance of high and low hybrid discriminations across measures. Two hybrid groups, highest and lowest 25th percentiles determined based on several variables: CHI_AUC_, CHI_FPCA_ (including both FPCA1 and FPCA2), grain yield (t/ha), and temporal NGRDI scores at each of 16 time points (given as days after planting, DAP) from irrigated and nonirrigated trials in Texas in 2020. These variables calculated in Texas in 2020 were used to assess their discrimination ability for the 2 hybrid groups across multiple environments and tester. a) Comparison of high and low percentile hybrid groups across the variables shown on the *x*-axis. Each cell represents a comparison between the 2 hybrid groups within each environment and tester combination. Red cells indicate significant differences (*P* < 0.10) between the high and low percentile groups, while blue cells indicate nonsignificant differences (*P* ≥ 0.10). This panel highlights the discriminability of hybrids with high and low CHIs based on the 4 variables. b) Temporal analysis of NGRDI values across 16 time points (days after planting, DAP), calculated based on irrigated and nonirrigated environments in Texas 2020. Each cell represents a *P*-value comparison between the high and low percentile hybrid groups at a specific DAP for each environment and tester combination. Red cells denote significant differences (*P* < 0.10), while blue cells denote nonsignificant differences (*P* ≥ 0.10). This illustrates the stability and robustness of the CHI in differentiating high- and low-performing hybrids across multiple time points and environmental conditions. Numbers at the top of each cell indicate the number of times the comparison was statistically significant based on *P* < 0.10.

The main CHIs, CHI_AUC_, and CHI_FPCA1_, along with grain yield and 16 temporal NGRDI scores (a total of 19 variables), were used as indices, each calculated to test separately from irrigated and nonirrigated trials in Texas 2020 using the PHZ51 tester (Fig. 5, a and b). These 19 indices from both irrigated and non-irrigated conditions were subsequently applied across multiple environments and testers to assess their ability to distinguish between high- and low-yielding hybrid groups.

The results indicated that indices derived from the favorable environment (irrigated trial) showed greater discriminative ability than those derived from the unfavorable environment (nonirrigated trial) in distinguishing between high- and low-yielding hybrids. Specifically, CHI_AUC_ and CHI_FPCA1_ calculated from the irrigated trial (TXH1.2020) achieved significant discrimination in 27 and 28 out of 41 environment–tester combinations, respectively (Fig. 5a). Only the irrigated trial was therefore used in further analyses.

In contrast, using grain yield (t/ha) from the irrigated trial (TXH1.2020) as an index resulted in less consistent discrimination across environments, with successful separation observed in only 21 combinations (Fig. 5a). Additionally, NGRDI values measured during the reproductive stage—particularly at 82 and 102 DAP in the irrigated trial (TXH1.2020)—achieved significant discrimination in 28 environment–tester combinations (Fig. 5b).

### Predictive performance of phenomic-environment index aided genomic prediction

**Figure 6:**
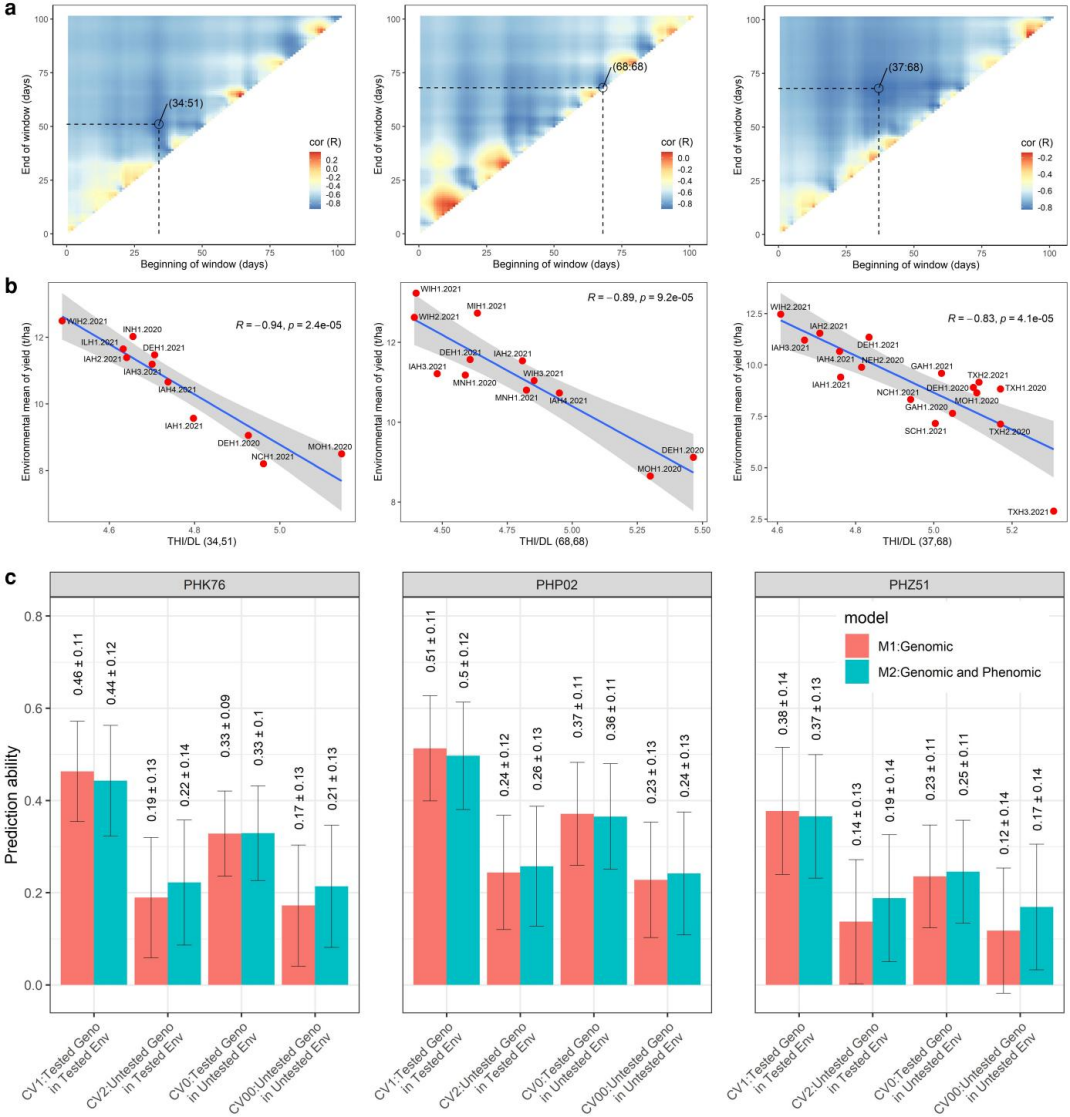
Environment data modeling and prediction accuracy across phenomic–environmental index-driven genomic prediction models. a) Heatmaps showing the correlation (cor) between the environmental index (temperature–humidity index divided by day length, THI/DL) and mean yield across environments over varying day intervals for each tester using all available data. The strongest correlations are marked with dashed lines, indicating the day intervals that most strongly influenced yield: (34 to 51) for PHK76, (68 to 68) for PHP02, and (37 to 68) for PHZ51. b) Linear regression plots showing the relationship between the mean environmental index (THI/DL), averaged across the selected critical day intervals, and mean grain yield across environments for each tester. Each point represents an individual environment (location × year). The line indicates the fitted linear regression, with the corresponding correlation coefficient (R) and *P*-value shown within each panel. The shaded area represents the 95% confidence interval around the regression line. Strong negative correlations (R values) indicate that environmental conditions during these specific time windows substantially affect yield. c) Bar plots show prediction abilities for models M1 (Genomic) and M2 (Genomic and Phenomic) across 4 prediction scenarios (CV1, CV2, CV0, and CV00) for each tester (PHK76, PHP02, PHZ51). The environmental index was used to define genotype-specific reaction-norms by estimating slopes and intercepts, which were subsequently predicted under M1 and M2 and used to reconstruct grain yield across environments. Error bars represent standard errors calculated across 100 iterations of the prediction models. M2 generally achieved higher prediction accuracy than M1, particularly in challenging scenarios such as CV2 and CV00.

The phenomic-environment index-driven genomic prediction models, M1 (Genomic) and M2 (Genomic and Phenomic), showed varying predictive abilities across different prediction scenarios and testers (Fig. 6, a to c). In each prediction scenario, M2, which incorporated a phenomic relationship matrix (PRM) derived from NGRDI values collected at 16 time points (flights) from an irrigated trial (TXH1.2020), consistently demonstrated improved prediction abilities compared with M1, particularly under challenging conditions such as CV2 (untested genotypes in tested environments) and CV00 (untested genotypes in untested environments) (Fig. 6, a to c).

An environmental index, THI/DL, was calculated using training data and successfully modeled environmental effects, providing positive prediction accuracy in both M1 and M2 across all prediction scenarios. When all data is considered, specific day intervals—34:51 for PHK76, 68:68 for PHP02, and 37:68 for PHZ51—captured critical environmental influences on yield, contributing to the improved prediction ability observed in both models. This approach demonstrated robustness across 3 different maize populations (PHK76, PHP02, and PHZ51), each grown in distinct locations across the United States, underscoring the generalizability and effectiveness of this environmental index in phenomic-environment index-driven genomic prediction (Fig. 6, a to c).

### Genomic region linked to temporal NGRDI trajectories

QTL intervals were prioritized, and candidate genes within these regions were explored for their potential roles in plant fitness during growth under both irrigated and nonirrigated conditions. Specifically, QTL mapping was performed for temporal NGRDI trajectories, with NGRDI values at 16 individual time points used as phenotypes in both irrigated and nonirrigated trials (Fig. 2c).

**Figure 7:**
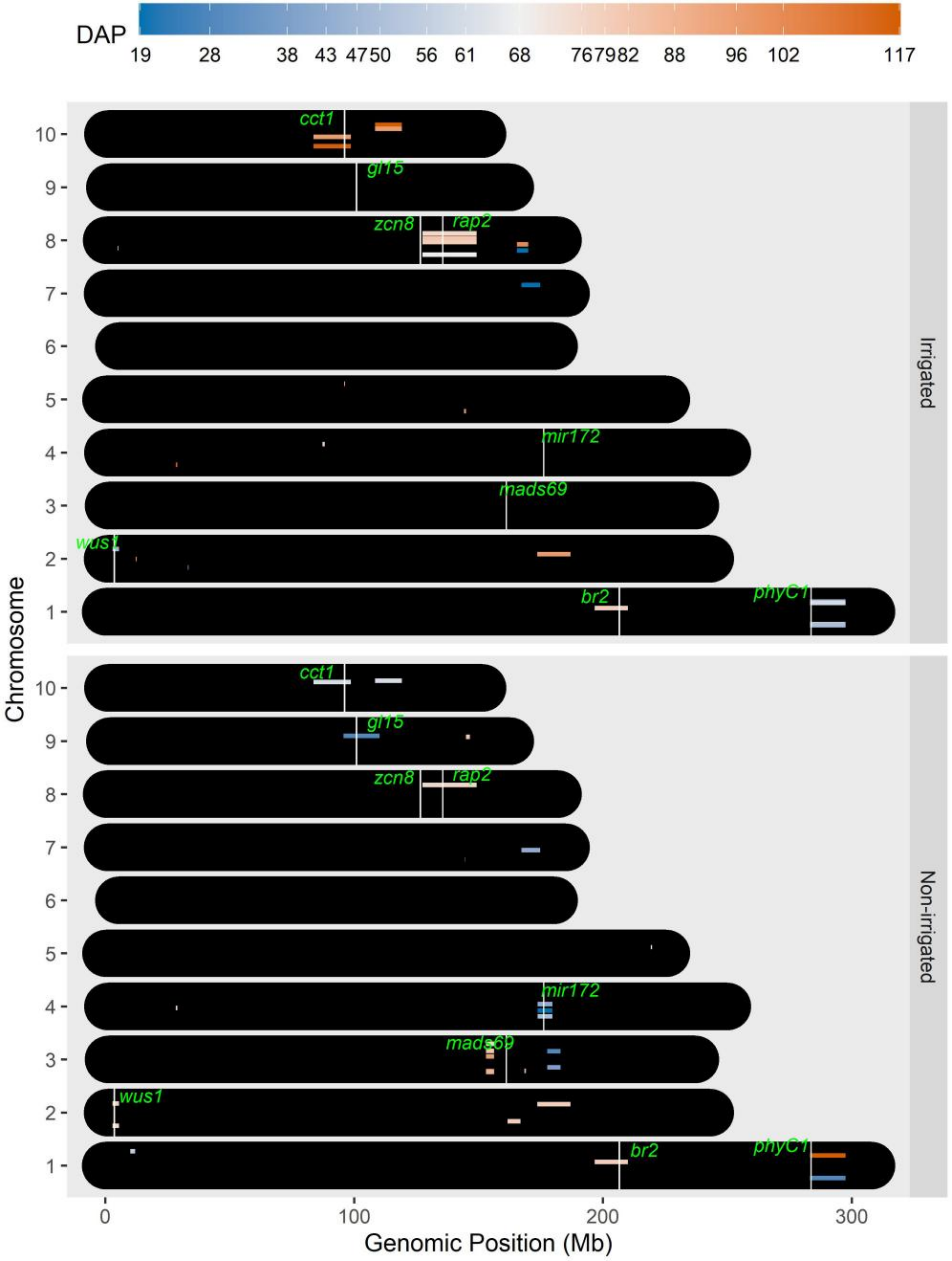
Genomic regions associated with NGRDI phenotypes identified through QTL mapping across irrigated and nonirrigated conditions. Horizontal lines represent the QTL intervals, with candidate genes indicated by white-labeled vertical lines and gene names shown in green. The colors of the QTL intervals correspond to the days after planting (DAP) at which they were detected, as indicated by the color scale at the top (reflecting UAV flight time points). The 2 panels separate QTL results for irrigated (top) and nonirrigated (bottom) conditions, with chromosomes arranged vertically from 1 to 10.

On chromosome 1, two significant intervals were identified. The first spans positions 196.7 to 210.1 Mb and is associated with both nonirrigated (DAP 79) and irrigated (DAP 79) trials. The second interval, spanning positions 283.3 to 297.6 Mb, is associated with irrigated trials at DAP 47, 50, 56, and 61, as well as nonirrigated trials at DAP 28 and 117. On chromosome 2, a QTL interval spanning 2.97 to 5.58 Mb is linked to both irrigated (DAP 47) and nonirrigated trials (DAP 76 and 79). Chromosome 3 revealed an interval from 152.99 to 156.28 Mb associated with nonirrigated trials at DAP 76, 79, 82, 88, and 96 (Fig. 7).

On chromosome 4, an interval spanning 173.81 to 179.68 Mb is linked to nonirrigated trials at DAP 19, 43, and 50. Chromosome 8 harbors a QTL interval spanning 127.46 to 149.29 Mb, associated with both trials at DAP 76 and with the irrigated trial at DAPs 68, 79, and 82. On chromosome 9, an interval spanning 95.70 to 110.29 Mb is linked to nonirrigated trials at DAP 28. Lastly, chromosome 10 features an interval from 83.75 to 98.77 Mb, associated with both irrigated (DAP 96 and 117) and nonirrigated (DAP 61) trials (Fig. 7).

## Discussion

HTFP, integrated with temporal analytical tools such as AUC and FPCA, provides a powerful approach for dissecting longitudinal crop health patterns and genotype-by-environment interactions. HTFP enables high-resolution temporal imaging of crops under diverse stress conditions, generating detailed temporal data that reflect genotype responses throughout the growth cycle. The AUC and FPCA metrics use this longitudinal data to provide a health index related to plant growth and resilience, offering a comprehensive assessment of plant performance over time while uncovering critical patterns of variation linked to genetic contributions of plant fitness. This study demonstrates how (i) the genetic mechanisms constructing crop resilience can be discovered by analyzing temporal phenotypic patterns; (ii) high- and low-yielding hybrids can be distinguished using HTFP data from a single low-yielding stress environment (like Texas), with these insights successfully applied to predict hybrid performance across multiple environments; (iii) yield prediction can be performed with enhanced precision by integrating multi-omics data with temporal phenomic, genomic, and environmental inputs, even in challenging predictive plant breeding scenarios (eg, untested genotypes in tested and untested environments).

### HTFP-derived CHIs differentiate hybrid yields and predict multi-environment performance

Crop health is best understood as cumulative and temporal response to diverse abiotic and biotic stressors throughout the plant growth cycle, encompassing various strategies such as stress avoidance, tolerance, and recovery (Kissoudis et al. 2014). This study underscores the utility of HTFP and advanced analytics in evaluating these multifaceted growth strategies, providing insights into how crops maintain productivity under stress while navigating health dynamics (Singh et al. 2016, 2021; Herr et al. 2023).

Because PHZ51 was used as the tester in the initial environment to calculate the CHIs, we would expect that it would provide the best separation between upper and lower grain yield. However, better separation was observed for PHK76. It is important to note that the Wisconsin Stiff Stalk MAGIC inbred population was created from stiff stalk germplasm and all 3 testers are nonstiff stalks (Michel et al. 2022). Therefore, these indices are detecting variation of the additive general combining ability across the inbred population lines, rather than specific combining ability across testers.

A major advancement of this finding is it suggests a measure of crop health from a single environment could be agnostic to the specific tester used to create the hybrid and could be used to predict performance across a very diverse set of environments. If this result is consistent across future studies, it will be of significant impact. It would also suggest that the number of multi-locational yield trials needed in earlier generations could be substantially reduced in plant breeding where temporal HTFP experiments are conducted. Multi-locational yield trials are among the largest expenses of plant breeding programs and are conducted to ensure stable performance across environments. Even if the efficiency of early-stage selections based on high throughput phenotyping data from limited environments is validated, multi-locational yield trials would still be needed for advanced-stage trials to demonstrate farmer and commercial relevance.

The findings of this study demonstrate that CHIs, specifically CHI_AUC_ and CHI_FPCA1_, derived from temporal phenomic data collected in a single favorable environment (irrigated trial in TXH1.2020), outperformed using grain yield from the same environment as a proxy in differentiating high- and low-yielding hybrids across multiple environments. While grain yield per se achieved significant differentiation only 21 times out of 41 environment-tester combinations, CHI_AUC_ and CHI_FPCA1_ succeeded 27 and 28 times, respectively (Fig. 5). This highlights the greater predictive power of temporal indices over static yield measurements, even when all metrics were derived from the same single favorable environment. Several key mechanisms explain this phenomenon. First, CHI_AUC_ and CHI_FPCA1_ integrate cumulative plant health responses captured across multiple time points during growth, providing a season-long perspective on plant development rather than a static endpoint like grain yield (Araus et al. 2018). This cumulative nature explains their ability to better differentiate hybrids across environments (Adak et al. 2023). Second, temporal indices are less susceptible to environmental noise since they average plant responses over key developmental stages rather than relying solely on yield, which is highly sensitive to stress factors encountered near harvest (White et al. 2012). This reduces the influence of random environmental variation on prediction accuracy.

Third, the predictive power of temporal indices derived from a single environment stem from their ability to map complete health trajectories. Previous research has shown that time-series phenotypic data can uncover genetic potential (Adak et al. 2024; Sweet et al. 2024); this characteristic makes temporal indices transferable across environments.

Additionally, the reproductive stage, particularly around 82 and 102 DAP, represents critical periods for biomass accumulation and grain filling. Temporal indices computed during these stages likely captured essential health dynamics, contributing to their superior predictive accuracy (Egli 2004; Reynolds et al. 2009).

Finally, using a favorable environment for data collection strengthens genetic signal detection by minimizing confounding stress-related variation. This approach aligns with a breeding strategy emphasizing selection in optimal conditions to improve overall yield resilience (Sinclair 2011).

### Multi-omic data integration strengthens yield prediction models through genomic, phenomic, and environment layers

Traditional genomic prediction models often assume stable genotype-environment interactions, limiting their ability to account for environmental variability (Heslot et al. 2014; Tibbs-Cortes et al. 2024). This assumption reduces predictive accuracy, especially when new genotypes or environments are introduced. In contrast, our approach dynamically captured environmental variation by calculating an environmental index based on the temperature-humidity index (THI) divided by day length (DL), following (Li et al. 2018). To avoid data leakage, the environmental index and its optimal day interval were recalculated using only the training dataset in each cross-validation iteration (ie, using only the tested hybrids and environments within the training fold). To further enhance model performance, we integrated a PRM derived from UAV-based temporal crop health data collected under a single environment (TX irrigated trial with PHZ51 tester). Although derived from a single environment, the PRM captures key dynamic phenomic traits indicative of plant health, which are critical for yield determination. Inclusion of the PRM was intended to provide complementary biological insight beyond static genomic markers, offering an additional layer of genotype-by-environment-by-phenotype (G × E × P) interactions. This addition evaluates whether phenomic signatures reflecting temporal plant health—despite originating from 1 environment—can enhance prediction accuracy across diverse and unseen environments, particularly under the more challenging CV0 and CV00 scenarios (Fig. 6, a to c).

While the final selected day intervals—34:51 for PHK76, 68:68 for PHP02, and 37:68 for PHZ51—were calculated using the full dataset for consistency and interpretation, these intervals were generally well captured from within the training data alone during model fitting. This demonstrates that the critical environmental windows contributing to prediction accuracy are identifiable without requiring access to the test environments, supporting the robustness of the approach.

Prediction ability was evaluated within each test environment, based on the correlation between observed and predicted yields. This approach ensured that the model ranked genotypes effectively within environments—a key goal for selection—rather than merely predicting environment means. By integrating THI/DL-based environmental indices into model training, we improved G × E modeling and enhanced within-environment prediction accuracy.

The strategy was successfully applied across 3 diverse maize populations (PHK76, PHP02, and PHZ51), each grown in different U.S. environments, reinforcing the generalizability of this phenomic–environment index–genomic prediction framework.

The integration of temporal NGRDI-derived PRM with genomic data significantly increased yield prediction accuracy across diverse environments. However, we acknowledge that a key limitation of this study is that the PRM was constructed using phenomic data from a single season and environment (TXH1.2020). While this result demonstrates the potential of phenomic–genomic integration, reliance on a single environment may not fully capture the breadth of genotype-by-environment-by-phenotype (G × E × P) interactions encountered across years and locations.

PRM was constructed using phenomic data from only 1 environment (TXH1.2020), and its inclusion in the prediction model enhanced yield prediction through a multi-omics approach by combining phenomic, genomic, and environmental index data (Zagorščak et al. 2025). The temporal nature of NGRDI enabled the PRM to capture key plant health dynamics closely linked to grain yield (Adak et al. 2024; Yan et al. 2024). When combined with genomic markers representing the genetic potential of genotypes, the PRM provided an additional layer of environmental adaptability by reflecting genotype-by-environment-by-phenotype (G × E × P) interactions, capturing how plant health dynamically responds to environmental stimuli. This multi-omics integration generalized predictions beyond the training environment, demonstrating that even a single-environment PRM can enhance predictive performance across mega-environments in multi-environment trials.

While PRM development from a single environment yielded promising results, expanding the phenomic dataset by integrating temporal data from additional environments would likely improve prediction accuracy even further. This approach would better represent G × E × P interactions by capturing a broader range of phenotypic responses across various climatic and management conditions (Adak et al. 2023). With multiple temporal phenomic datasets from contrasting environments, the PRM could more accurately reflect the dynamic responses of genotypes, boosting the precision of yield prediction across diverse environments. This scalability makes phenomic-environmental data integration a promising tool for advancing prediction models in plant breeding programs.

### Temporal data reveals the time-sensitive genetic interplay driving crop fitness

To hint at underlying biological causes, temporal NGRDI was used as the phenotypic trait for QTL analysis, capturing plant health dynamics across 16 drone-based observation time points under both irrigated and nonirrigated conditions in Texas. While this temporally dense phenotyping enabled the detection of time-dependent genetic signals related to growth, the analysis was conducted within a single growing season and location. As plant growth trajectories are strongly influenced by environmental conditions, the identified QTLs reflect genetic responses specific to the conditions evaluated there. Ongoing and planned phenomic data collection across multiple environments and time points will enable validation of these loci and assessment of their stability and broader relevance. Candidate genes within the detected QTL intervals were selected based on positional overlap and biological relevance to growth and stress responses and are therefore proposed as plausible contributors rather than confirmed causal determinants, pending functional validation and confirmation using multi-environment temporal datasets.

On chromosome 1, two QTL intervals were identified. The first interval (196.7 to 210.1 Mb) was associated with canopy greenness at 79 DAP under nonirrigated conditions and contains *br2*, which is proposed as a candidate gene based on its established role in auxin-mediated plant architecture (Multani et al. 2003; Zhang et al. 2019). A second interval on chromosome 1 (283.3 to 297.6 Mb) was detected at DAPs 28, 47, 61, and 117 across both irrigated and nonirrigated trials and harbors *phyC1*, a gene involved in light-response regulation (Neff et al. 2000).

On chromosome 2, a QTL interval spanning 2.97 to 5.58 Mb was identified at DAPs 47, 76, and 79 in both management conditions and includes *WUS1*, a gene implicated in shoot apical meristem maintenance and organogenesis under variable environments (Chen et al. 2021). On chromosome 3, a QTL detected exclusively under nonirrigated conditions at DAPs 76, 79, 82, 88, and 96 mapped to an interval (152.99 to 156.28 Mb) located approximately 5 kb from *MADS69*, which is therefore considered a candidate gene based on its role in flowering regulation via the *ZmRap2.7–ZCN8* pathway (Liang et al. 2019).

A QTL on chromosome 8 (127.46 to 149.29 Mb), detected at DAPs 68, 76, 79, and 82 under irrigated conditions, encompasses *RAP2.7*, a known regulator of photoperiod-sensitive flowering through modulation of *ZCN8* expression (Salvi et al. 2007). On chromosome 10, a QTL interval spanning 83.75 to 98.77 Mb was detected at DAPs 61, 96, and 117 across both trials and contains *ZmCCT10*, which is proposed as a candidate gene due to its documented roles in photoperiod response and stress adaptation (Huang et al. 2018; Su et al. 2021).

Finally, QTL intervals on chromosome 4 (173.81 to 179.68 Mb) and chromosome 9 (95.70 to 110.29 Mb), identified at early and mid-season DAPs under nonirrigated conditions, include *miR172* and *gl15*, respectively. These genes are considered plausible candidates given their involvement in vegetative phase transition and developmental plasticity under stress conditions (Lauter et al. 2005).

These findings demonstrate the effectiveness of HTFP and multi-time-point temporal data in dissecting complex genotype-environment interactions. Temporal NGRDI measurements captured dynamic plant responses, enabling the detection of environmentally responsive QTLs and key candidate genes. This integrative approach offers a promising pathway for unraveling the genetic basis of crop development and fitness, facilitating precision breeding for resilient and resource-efficient maize varieties. The following postulated genetic mechanisms highlight the broader implications of this study:

- Genetic Regulation of Canopy Fitness: The detection of potential candidates *br2* and *phyC1* would suggest auxin-mediated and light-responsive regulatory pathways influencing canopy development and greenness under stress conditions (Neff et al. 2000; Multani et al. 2003). These genes provide targets for enhancing light capture and photosynthetic efficiency.
- Meristem Stability and Organogenesis: The identification of potential candidate *wus1* under both irrigation regimes would highlight shoot apical meristem maintenance, promoting consistent organogenesis and canopy development through critical stages (Chen et al. 2021). This supports breeding efforts for enhancing plant vigor and structural resilience.
- Flowering Resilience under Drought: The exclusive detection of a QTL containing potential candidate *mads69* in nonirrigated trials would underscore its role in modulating flowering adaptation under water-limited conditions. Its upstream regulation of potential candidates *rap2-zcn8* would highlight a genetic mechanism connecting flowering resilience to drought adaptation (Liang et al. 2019).
- Photoperiod Sensitivity and Environmental Adaptation: Potential candidates *rap2* and *cct1* linked to both irrigated and nonirrigated trials would highlight a critical regulatory axis managing photoperiod sensitivity, flowering suppression, and maintaining development even in drought-prone trials (Salvi et al. 2007; Huang et al. 2018; Su et al. 2021).
- Plasticity and Stress Tolerance: Potential candidates *miR172* and *gl15* associated with phase-change regulation would suggest involvement in extending vegetative growth and delaying flowering under drought conditions. This regulatory interplay could be critical for enhancing maize resilience and plasticity (Lauter et al. 2005).

Overall, these postulated genetic mechanisms, suggested through precise phenotyping and temporal data analysis, would provide a robust framework for exploring and manipulating resilience-associated genetic pathways if confirmed. The combination of drone-based temporal measurements, multi-environment trials, and genetic mapping represents a scalable and efficient approach to improving maize productivity, adaptability, and resilience in diverse agricultural systems.

## Conclusion

HTFP using temporal analytics such as AUC and FPCA, provide a robust framework for assessing crop health trajectories through season-long performance monitoring. This study demonstrated that (i) temporal indices derived from NGRDI data effectively captured cumulative health responses across critical developmental stages, enabling an in-depth evaluation of maize performance; (ii) QTL containing key candidate genes linked to temporal NGRDI, including *br2*, *phyC1*, *wus1*, *mads69*, *cct1*, *rap2*, *miR172*, and *gl15*, were identified, suggesting genetic mechanisms regulating maize fitness; (iii) predictive power from a single environment was achieved, as temporal indices differentiated high- and low-yielding hybrids across diverse environments, demonstrating environmental transferability; (iv) multi-omics data integration strengthened prediction models by combining phenomic, genomic, and environmental inputs, enhancing the precision in grain yield prediction in multi-locational trials; and (v) scalable breeding applications emerged from these findings, offering a data-driven framework for developing climate-resilient and resource-efficient maize varieties adaptable to diverse agricultural settings.

## Materials and methods

### Population type, experimental design, and trial

The 250-hybrid population used consisted of 2020 Genome to Fields project maize germplasm (Lima et al. 2023). Specifically 220 hybrids employed were generated by crossing a set of doubled-haploid inbred lines sourced from the Wisconsin Stiff Stalk MAGIC population (Michel et al. 2022) with PHZ51, a nonstiff stalk tester. These hybrids were grown and phenotyped in both irrigated and nonirrigated trials conducted in 2020 at the AgriLife Research farm near College Station, Texas. Both trials were planted on 11 March 2020. In each trial, entries were arranged in a modified randomized complete block design with 2 replications. Each hybrid was planted in a 2-row plot in each replication. The 2-row experimental plots were standardized to a length of 7.62 m, and rows were spaced 0.76 m apart.

### High throughput field phenotyping: drone, sensor, image processing, and data extraction

RGB format images were captured using a DJI Phantom 4 Pro V2.0 equipped with a 1-inch 20MP CMOS sensor featuring a mechanical shutter (created by SZ DJI Technology Co., Ltd., Shenzhen, China). The flight missions, managed through the DJI GS Pro application, were conducted at a height of 25 m above the ground with 90% forward and 80% side overlaps. Each RGB image had a ground sampling distance of 0.7 cm/pixel. Each RGB flight encompassed both the irrigated and nonirrigated trials concurrently. UAV flights were conducted on 16 different Days: 19, 28, 38, 43, 47, 50, 56, 61, 68, 76, 79, 82, 88, 96, 102, and 117 DAP (Fig. 1, a and b). Agisoft Metashape software was utilized first to process the images within each flight.

Orthomosaics were produced by combining the software's structure-from-motion technique with multi-view stereo methodology. Ground control points were established using a V-map Dual Frequency L1/L2 PPK GNSS Receiver from Micro Aerial Projects. The orthomosaic creation workflow included: loading raw RGB images into an Agisoft project, setting the coordinate system and projection (usually WGS84), aligning photos, conducting an initial bundle adjustment to optimize camera calibration parameters, enhancing tie point accuracy, importing ground control points, aligning images, adjusting the bounding box, generating a dense cloud, color calibration, and creating a digital elevation map.

Normalized Green Red Difference Index (NGRDI; $\frac{\mathrm{Green}\text{-}\mathrm{Red}}{\mathrm{Green}+\mathrm{Red}}$) vegetation index was extracted for each 2-row plot from RGB orthomosaics (Tucker 1979). NGRDI was selected because it is a robust and cost-efficient RGB-based index that is sensitive to canopy greenness and vegetation vigor and has been reported to be associated with maize grain yield; supporting its use for tracking temporal variation in overall plant health across growth and for downstream analyses, including QTL discovery and grain yield prediction in maize (Adak et al. 2021, 2024). The R/UAStools package was used to create the plot-based shapefiles (Anderson and Murray 2020). Then, orthomosaics and shapefiles were used in R/FIELDimageR to calculate the NGRDI values for each 2-row plot (Matias et al. 2020). Soil was masked to avoid confounding canopy coverage with plant health in this index. As a result of these processes, NGRDI values were obtained from a total of 16 flights, resulting in a raw dataset comprising 16,000 data points (250 hybrids × 2 managements × 2 reps × 16 flights).

### Statistical evaluation of time-series NGRDI

In the previous section, plot-based raw temporal data with NGRDI was generated for the hybrids. These raw data were analyzed using the statistical model (Eq. 1) for temporal data analysis as follows:


(1) $$V_{iklm}=\mu +\gamma_{i}+\lambda_{k}+(\gamma \lambda {)}_{ik}+f_{lm}(col,row)+\varepsilon_{iklm}$$

Where, $V_{iklm}$ represents the observed plot-based NGRDI value for the i−th hybrid genotype, under the k−th management (irrigated or nonirrigated), at the l−th row and m−th column in the field, for each of the 16 flight time points. *μ* is the overall mean NGRDI, $\gamma_{i}$ is the random effect of the i−th hybrid (genotype), assumed to be distributed as $\gamma_{i}\;\underset{∼}{IID}\;N(0,\sigma_{\gamma }^{2})$. $\lambda_{k}$ is the fixed effect of the k−th management (k=irrigated,non-irrigatedtrials), and $(\gamma \lambda {)}_{ik}$ represents the genotype by management interaction effect. $f_{lm}(col,row)$ represents the 2-dimensional smoothing with P-splines function of the column number (*l*) and row number (*m*) of the field, capturing the spatial effect, modeled using the *SpATS::PSANOVA()* function of SpATS in R. $\varepsilon_{iklm}$ is the residual error, assumed to be distributed as $\;\varepsilon_{iklm}\underset{∼}{IID}\;N(0,\sigma_{\varepsilon }^{2})$.

The model was run for each flight time point using statgenHTP package in R (https://cran.r-project.org/web/packages/statgenHTP/), which includes the SpATS package (Rodríguez-Álvarez et al. 2018). The statistical model was run using the *statgenHTP::fiModels()* with *geno.decomp=“management”* to include the management effect as fixed and to estimate the variance decomposition for the hybrids at each management level in each time point. Heritability was calculated for each time point in both management conditions using the *statgenHTP::getHerit()* function.

### Crop health index calculation

In this study, 2 CHIs were calculated using the temporal NGRDI values collected from hybrids in the flights conducted at 16 flight times from 19 to 117 DAP (Fig. 1a). These flight times cover the entire growth stages of hybrids (Fig. 1, a and b).

The first crop health index, termed CHI_AUC_, was derived by calculating the area under the temporal NGRDI curve for each hybrid. Specifically, we used the auc() function from the MESS R package (*MESS::auc()*), which numerically integrates a curve formed by time-series data. In our case, the curve consisted of 16 NGRDI values collected at different time points during the growing season. To construct a smooth trajectory between these discrete points, we applied the *type = “spline”* option within the function, which fits a natural cubic spline interpolation across the time series. The area under this interpolated curve—representing the hybrid's cumulative spectral health response over time—was separately computed using the trapezoidal rule, which approximates the integral of the curve between the consecutive time points. The cubic spline and trapezoidal calculations were functionally equivalent so cubic spline was used. Cubic spline interpolation was used only to facilitate numerical integration for AUC calculation and was not used for smoothing or modeling temporal NGRDI trajectories in downstream genetic or prediction analyses.

This calculation was performed separately for each hybrid under both irrigated and nonirrigated conditions. The resulting AUC values were designated as the CHI_AUC_ scores, representing cumulative health intensity under contrasting water regimes (Fig. 1c).

The second crop health index, termed CHI_FPCA_, was derived using FPCA, a technique that captures dominant patterns of variation in temporal traits (Miao et al. 2020; Adak et al. 2024). We used the FPCA() function from the fdapace R package (*fdapace::FPCA()*), which decomposes time-series data into orthogonal basis functions representing major modes of temporal variation. For each hybrid genotype, we input the NGRDI values observed across 16 UAV flight time points, along with their associated DAP.

Since the time-series data consisted of irregular and sparsely collected observations across hybrids, we specified *dataType = “Sparse”* within the function to model these FPCA trajectories appropriately under a sparse functional data framework. This allowed the function to estimate the mean function and eigenfunctions from the full set of sparse time points across the population, and then calculate individual-level FPCA scores (eg, FPCA1 and FPCA2) for each hybrid. These scores summarize the shape and magnitude of each genotype's health trajectory over time.

To determine the CHI_FPCA_, the first 2 FPCA components—FPCA1 and FPCA2—were extracted separately for each hybrid under both irrigated and nonirrigated conditions, and used as composite indicators of temporal health dynamics (Fig. 1d).

Here, 2 specific measures of CHIs— CHI_AUC_ and CHI_FPCA_—were introduced and subsequently utilized as phenotypes calculated and associated with grain yield across many environments.

### Relationship between CHIs and grain yield

In addition to the field trial in Texas, hybrids derived from the same inbred population crossed with 3 testers—PHZ51, PHK76, and PHP02—were grown in 25 unique environments across the United States during 2020 and 2021 as part of the G2F G×E Project (Lima et al. 2023; Ozair et al. 2025).

To get the genotypic effects for grain yield (t/ha), Equation 1 was applied without the management effect ($\lambda_{k}$), focusing solely on genotype ($\gamma_{i}$) and spatial factors (row and column). This simplified model was run separately for each of 25 environments. The multi-environment yield data was then used to integrate with CHI_AUC_ and CHI_FPCA_, allowing for the exploration of associations between genotypic performance and health dynamic across different environments. To achieve this association, the grain yield values of hybrids in the upper and lower 25th percentiles of CHI_AUC_ and CHI_FPCA_, calculated from both the favorable (irrigated trial TXH1.2020) and unfavorable (nonirrigated trial TXH2.2020) environments, were selected, and 2 groups were created accordingly. These 2 groups—hybrids in the upper and lower 25th percentiles based on grain yield—were initially compared using the Wilcoxon test (*P* < 0.10) across 25 environments to evaluate whether the CHIs (CHI_AUC_ and CHI_FPCA_) effectively differentiated high- and low-yielding genotypes outside of the UAV data collection environment. To further investigate the stability and performance of these groups across environments, we conducted separate linear regressions for each group: the mean grain yield of the upper percentile group was regressed on the mean yield of the environment, and likewise for the lower percentile group. This allowed us to assess whether higher-performing genotypes showed greater yield potential and stability (via regression slope and intercept) as environmental conditions changed. Importantly, this regression analysis was performed independently for each tester, as the associated hybrid sets and trial environments differed.

### Environmental characterization by multivariate analysis

Environmental conditions across trials were characterized using a temporally explicit environment index derived from daily weather observations. Many indices were explored from the available data, however they were also highly correlated and provided little difference, and increased complexity when used jointly. Therefore we chose one that had the highest explanatory power and captured the main independent factors of heat and DL. For each environment *e* and day *d*, an environmental index was defined as


$$EI_{e,d}=\;\frac{THI_{e,d}}{DL_{e,d}},$$

where $THI_{e,d}$ denotes the daily thermal heat index and $DL_{e,d}$ represents day length (Yousef 1985). The index was calculated for each day from planting (d=1) to 100 days after planting (d=100), corresponding to the main vegetative and early reproductive growth phases. The resulting time series


$$\left\{EI_{e,1},EI_{e,2},\ldots ,EI_{e,100}\right\}$$

provides a compact representation of temporal environmental conditions for each environment.

Daily index values were arranged into a matrix


$$E\in R^{E\times D},$$

where *E* is the number of environments and D=100 is the number of days from planting within each environment. To summarize dominant temporal patterns while preserving the functional nature of the data, FPCA was applied under a sparse-data framework. Let $X_{e}(t)$ denote the continuous environmental trajectory for environment *e*. FPCA decomposes this trajectory as


$$X_{e}(t)=\;\mu (t)+\overset{K}{\underset{k=1}{\sum }}\;\xi_{e,k}\;\phi_{k}(t)+\varepsilon_{e}(t),$$

where μ(t) is the mean environmental function, $\phi_{k}(t)$ are orthonormal eigenfunctions, $\xi_{e,k}$ are environment-specific FPCA scores, and $\varepsilon_{e}(t)$ is the residual error term. The first 3 functional principal components (K=3) were retained, capturing the majority of variation among environmental trajectories.

To further group environments with similar temporal profiles, k-means clustering was performed on the standardized matrix E. Prior to clustering, each column of E was centered and scaled to unit variance. The optimal number of clusters *k* was selected by maximizing the average silhouette width,


$$\bar{s}(k)=\;\frac{1}{E}\overset{E}{\underset{e=1}{\sum }}\;s_{e}\;(k),$$

where $s_{e}(k)$ denotes the silhouette width of environment eunder kclusters. Final cluster assignments were integrated with FPCA scores to facilitate visualization and interpretation of environmental similarity in reduced-dimensional space.

This framework was used exclusively to describe and contextualize the environmental diversity represented in the study and was not used directly for phenotypic or genetic modeling. Figure S1 shows the distribution of environments along with their k-means–based cluster assignments.

### Genotype by environment interaction

To calculate the percent variation explained by interaction between genotypes (maize hybrids) and environment, a conventional genotype by environment model was used to estimate the percent variation explained by genotype, environment, genotype by environment, and field spatial effects across multiple environments during 2020 and 2021 by Equation 2.


(2) $$y_{ijklm}=\mu +g_{i}+E_{j}+gE_{ij}+Column(E{)}_{k(j)}+Row(E{)}_{l(j)}+Rep(E{)}_{m(j)}+\varepsilon_{ijklm}$$

where, $y_{iklmn}$ is the plot-based grain yield (t/ha) data recorded by the plot-combine machine belonging to i−th hybrid, j−th environment, k−th column, l−th row and m−th replication. *μ* is the overall mean. $g_{i}$ is the random effect of i−th hybrid with $g_{i}\;\underset{∼}{IID}\;N(0,\sigma_{g}^{2})$. $E_{j}$ is the random effect of j−th environment with $E_{j}\;\underset{∼}{IID}\;N(0,\sigma_{E}^{2})$. $Column(E{)}_{k(j)}$ is the random effect of k−th column nested within j−th environment with $Column\;(E{)}_{k(j)}\;\underset{∼}{IID}\;N(0,\sigma_{Column}^{2})$. $Row(E{)}_{m(j)}$ is the random effect of m−th row nested within j−th environment with $Row(E{)}_{m(j)}\;\underset{∼}{IID}\;N(0,\sigma_{Row}^{2})$. $\varepsilon_{ijklm}$ is the residual error.

Heritability (*H*) for grain yield was calculated as follows: $H=\frac{\sigma_{g}^{2}}{\sigma_{g}^{2}+\frac{\sigma_{gE}^{2}}{a}+\frac{\sigma_{\varepsilon }^{2}}{ab}}$, where $\sigma_{g}^{2}$, $\sigma_{gE}^{2}$ and $\sigma_{\varepsilon }^{2}$ are the variances of genotype, genotype by interaction and error, and *a* and *b* are the number of environment and replications, respectively.

To reveal the variation driven by genotype-by-environment interaction ($gE_{ij}$), the traditional Finlay-Wilkinson regression approach was used by regressing the yield of each hybrid on the mean grain yield of environments within each tester category (Finlay and Wilkinson 1963).

### Phenomic and environment index driven genomic prediction approach

Traditional genomic prediction across multiple environments was improved by incorporating an environmental index and phenomic data. The steps for phenomic-environment index-driven genomic prediction were as follows: First, the data were split into 70:30, where 70% of the genotypes were treated as tested, and 30% as untested. Additionally, 1 environment was dropped and treated as an untested environment, while the remaining environments were used in the model as tested environments; models were trained using tested genotypes and environments. For each left-out environment, the 70:30 genotype partitioning was repeated for 100 independent iterations using different random splits, and prediction performance was summarized across these iterations. Second, the THI (Yousef 1985) was divided by DL for each day across all environments. Third, to determine which period of the growing season was most predictive of yield, the rolling correlation between the environmental index and the mean grain yield of each environment was computed across all possible time windows ranging from 1 to 100 d. For example, the correlation between the mean environmental index from Days 1 to 5 and the mean yield across environments was calculated; this process continued sequentially (eg, Days 2 to 6, 3 to 7, … , up to 96 to 100). Crucially, this correlation analysis was performed separately within each training iteration using only the tested genotypes and environments to avoid data leakage. In each iteration, the optimal time interval with the strongest correlation—either positive or negative—was selected for use in modeling. Fourth, a line was fit for each tested genotype across the mean environmental index of the day interval that provided the strongest correlation with the mean grain yield across environments (calculated in Step 3). At the end, the slope and intercept of this linear fit line were retrieved for each tested hybrid. Fifth, the model was trained to predict the slope and intercept separately for all hybrids. These predicted slopes and intercepts were then used to calculate the predicted yield at every environment, according to the formula: predicted yield = predicted slope * environment index + predicted intercept. Two different models were used to predict the slope and intercept separately for all hybrids. The first model (M1) contained the genomic relationship matrix (GRM) constructed from 252,286 segregating markers; genomic markers were explained here (Lima et al. 2023). The second model (M2) combined both the GRM and a PRM, which was derived from NGRDI values obtained from 16 UAV flights in a favorable (irrigated trial) environment (TXH1.2020). Both GRM and PRM were constructed using the rrBLUP::Amat() function in R, where marker and phenomic data were internally centered and scaled prior to matrix construction (Endelman 2011). Both models were run using BGLR in R (Pérez and de los Campos 2014). All these steps were conducted separately for each tester due to the differences in their growing environments.

To summarize how predictions were generated, genotype performance across environments were described using a reaction-norm framework. For each genotype *i*, the reaction-norm parameters consisted of an intercept $\beta_{0i}$ and a slope $\beta_{1i}$. Grain yield in environment *j* was predicted as:


$${\hat{Y}}_{ij}={\hat{\beta }}_{0i}+{\hat{\beta }}_{1i}\;EI_{j}$$

where $EI_{j}$ denotes the environmental index of environment *j*.

Under Model M1, the intercept and slope parameters were predicted using the genomic relationship matrix (GRM) only:


$$\beta_{0}=\mu_{0}1+u_{0},\;\;\;\;\;\;\;\;\;\;\;\;\;\;\;\;\;\;\;\;\;\;\;\;\;\;\;\;\;\;u_{0}∼N\;(0,\sigma_{0,g}^{2}K_{G})$$

$$\beta_{1}=\mu_{1}1+u_{1},\;\;\;\;\;\;\;\;\;\;\;\;\;\;\;\;\;\;\;\;\;\;\;\;\;\;\;\;\;\;\;u_{1}∼N\;(0,\sigma_{1,g}^{2}K_{G})$$

where

$\beta_{0}=(\beta_{01},\ldots ,\beta_{0n}{)}^{⊤}$ and $\beta_{1}=(\beta_{11},\ldots ,\beta_{1n}{)}^{⊤}$

are vectors of intercepts and slopes across genotypes, $K_{G}$is the GRM, and 1is a vector of ones.

Under Model M2, the intercept and slope were predicted using a multi-kernel formulation that jointly incorporated genomic and phenomic information:


$$\beta_{0}=\mu_{0}1+u_{0}+v_{0},\;\;\;\;\;\;\;\;\;\;\;\;\;\;\;\;\;\;\;\;u_{0}∼N\;(0,\sigma_{0,g}^{2}K_{G}),\;v_{0}∼N\;(0,\sigma_{0,p}^{2}K_{P})$$

$$\beta_{1}=\mu_{1}1+u_{1}+v_{1},\;\;\;\;\;\;\;\;\;\;\;\;\;\;\;\;\;\;\;\;u_{1}∼N\;(0,\sigma_{1,g}^{2}K_{G}),v_{1}∼N\;(0,\sigma_{1,p}^{2}K_{P})$$

where $K_{P}$ is the PRM derived from UAV-based NGRDI measurements. Thus, M1 represents a genomic-only reaction-norm model, whereas M2 extends this framework by incorporating phenomic information to improve the prediction of genotype-specific intercepts and slopes, which were subsequently used to predict grain yield across environments.

Finally, 4 prediction scenarios (CV1, CV2, CV0, and CV00) were calculated for both models (M1 and M2) by Pearson's correlation between the actual and predicted grain yield, each corresponding to different combinations of tested and untested genotypes in tested and untested environments: CV1: This refers to the correlation between actual and predicted grain yield for genotypes and environments that were already included in the training dataset. Rather than evaluating the model's predictive performance in unfamiliar contexts, this scenario quantifies how closely the model replicates the data it was trained on. It reflects the model's fit to the training set and indicates the degree to which the predicted values deviate from the observed values within the known data structure, offering insight into possible overfitting or underfitting. CV2: This scenario evaluates the model's capacity to predict the performance of previously unobserved genotypes in environments that were part of the training set. While the environmental conditions are familiar to the model, the genetic profiles of the untested hybrids are new. This setting closely mirrors a breeding selection context, where breeders aim to identify promising new genotypes under well-characterized environmental conditions. It tests the model's ability to generalize across unseen genetic backgrounds and provides insights into the genomic prediction accuracy for novel breeding lines. CV0: Here, the model predicts the yield of genotypes that were included in the training data, but in environments that were not used during training. This tests the model's environmental extrapolation ability and evaluates how well genotype-specific patterns can be transferred to new, previously unobserved environmental conditions. This scenario is particularly relevant in climate-resilient breeding programs, where known hybrids are evaluated for their performance in emerging or variable agroecological zones. It also helps quantify the stability and plasticity of genotypes under changing conditions. CV00: This represents the most rigorous and realistic evaluation of model generalizability. In this scenario, both the genotypes and environments are excluded from the training dataset, simulating a true deployment case where breeders must predict performance for new hybrids to be planted in new environments. This cross-validation setting reflects the highest level of uncertainty and is used to assess the model's robustness and reliability under complete novelty, which is critical for long-term predictive breeding strategies aimed at accelerating genetic gain in untested conditions.

#### Important note on M1 and M2 models

While the genomic prediction model (M1) relies solely on genomic data—which remains constant across different environments—the phenomic + genomic model (M2) incorporates phenomic features derived from UAV-based growth observations exclusively conducted under Texas irrigated conditions. Therefore, although the genomic data in M2 remains stable across environments, the phenomic variables, and thus the derived PRM, are specific to plant health behavior observed only in the TX irrigated trial from plants tested with the PHZ51 hybrid tester. These UAV-derived phenomic features were applied as additional predictors (kernels) across environments in the model, even though they originated from a single environment and tester combination.

It is critical to note that the PRM reflects health behavior captured solely under TX irrigated conditions and the PHZ51 tester background, and its contribution to improving yield prediction across different CV scenarios (CV1, CV2, CV0, and CV00) is explicitly tested in this study.

This approach allows for evaluating whether the integration of phenomic data can positively enhance prediction precision relative to genomic data alone, even under challenging scenarios involving unseen environments (CV0 and CV00), even if the phenomic data may not fully capture the full extent of environmental variation across all target environments as well as other testers in the full dataset (PHK76 and PHP02).

### QTL mapping for temporal NGRDI

A total of 100,000 SNPs were used in QTL mapping, evenly spanning all 10 chromosomes to suggest potential biological bases of the phenomena we observed. Data were from 220 inbreds that were crossed with PHZ51 tester and grown in both favorable (TXH1.2020; irrigated trial) and unfavorable (TXH2.2020; nonirrigated trial) environments in Texas, used in QTL mapping. For more information on the genomic data, refer to (Michel et al. 2022). NGRDI data from 16 time points, collected in both TXH1.2020 and TXH2.2020 environments in Texas, were used as phenotype data in QTL mapping to identify genomic regions influencing temporal NGRDI patterns. QTL mapping was conducted using R/qtl2, with the population type set to “dh6,” in line with the development design of the 6-way doubled haploid Wisconsin MAGIC population (Broman et al. 2019). The significance threshold for QTLs was determined using 1000 permutations with the R/qtl2::scan1perm() function, resulting in a LOD threshold of 5. Candidate genes within the significant regions were identified using the Zm-B73NAM-5.0 reference genome on MaizeGDB (https://jbrowse.maizegdb.org/).

## Supplementary Material

kiag344_Supplementary_Data

## Data Availability

https://github.com/alperadak/Phenomics-Enviromics-Genomic-Informed-Yield-Prediction-of-Maize
