# Supplementary material for: Phenomics-derived temporal maize health and environmental index enhance physiology-informed genomic prediction of yield across environments
Source: Plant Physiol. 2026 Jul 30;201(3):kiag344. doi: 10.1093/plphys/kiag344 (PMC13421785; doi:10.1093/plphys/kiag344)
Supplement: kiag344_Supplementary_Data [file kiag344_supplementary_data.pdf]

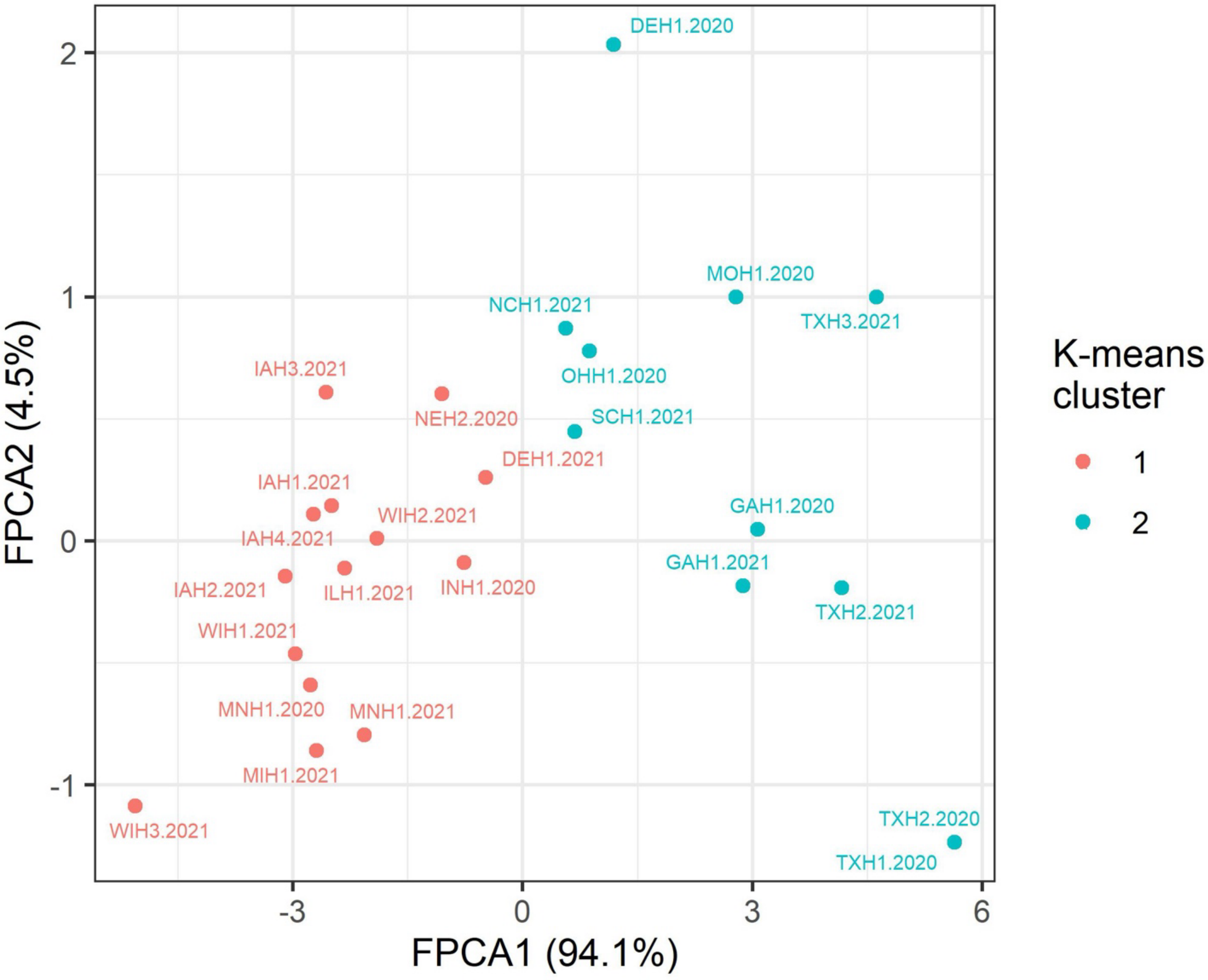

Supplementary Figure S1. Multivariate characterization of environments (location × year) used in downstream analyses and prediction modeling. Scatter plot of the first two functional principal components (FPCA1 and FPCA2) derived from the time-series environmental index (THI) calculated from planting to 100 days after planting. FPCA1 and FPCA2 explain 94.1% and 4.5% of the total temporal variation, respectively. Each point represents an environment defined as a location × year combination, included in downstream analyses and the prediction model. Colors indicate k-means cluster membership, grouping environments with similar temporal environmental profiles.
